# The Best Online Tools Based on Media Preference Reflected by Health Information Received on Social Media amongst Diabetic Patients in Hospital Canselor Tuanku Muhriz, Kuala Lumpur, Malaysia

**DOI:** 10.21315/mjms2021.28.3.11

**Published:** 2021-06-30

**Authors:** Hussein Siti Zuhaida, Hong Chuo Chung, Fawwaz Mohd Said, Khairunnisa Tumingan, Nurshalin Sahar Shah, Suraya Hanim, Abu Bakar

**Affiliations:** Nursing Department, Faculty of Medicine, Universiti Kebangsaan Malaysia, Kuala Lumpur, Malaysia

**Keywords:** media preference, diabetes, social media, Twitter, online search, health information

## Abstract

**Background:**

Diabetes mellitus has become a major public health problem globally. Social media could be useful in assisting clinical practice and sharing health-related information to improve self-management and to promote a positive behavioural change. This study aims to develop a guide on the best online tools by determining the media preference reflected by health-related information received from social media amongst diabetic patients in Hospital Canselor Tuanku Muhriz (HCTM), Universiti Kebangsaan Malaysia, Kuala Lumpur.

**Methods:**

This study was conducted cross-sectional on 174 respondents, who were selected by using a simple random sampling method. Socio-demographic data and the use of the internet and media for health-related information were obtained via questionnaires.

**Results:**

The most preferred social media used for searching and sharing health-related information was WhatsApp (73.6%), followed by Facebook (67.8%), Instagram (18.4%) and Twitter (17.2%). The social media preference related to socio-demographic data of age was statistically significant (*P* < 0.002), which had a medium effect. Furthermore, the media preference was not significantly related to health-related information searched or shared on social media and the frequency of usage.

**Conclusion:**

Indeed, the social media have been an essential media platform to enhance public awareness concerning public health. This calls for evolution to further enhance the use of social media amongst healthcare practitioners to emphasise health promotion and empower the patients to play an active role in their healthcare. This study provides a guideline for the medical researchers, practitioners or healthcare providers in choosing WhatsApp as an online medium to communicate with diabetic patients in the future, specifically in Malaysia.

## Introduction

Diabetes has been recognised as the most common disease amongst the public. Based on the International Diabetes Federation (1), there was about 463 million adults (20–79 years old) living with diabetes worldwide and this trend is estimated to increase to 700 million by the year 2045. In 2019, about 3,652,600 Malaysian adults were diagnosed with diabetes. Diabetes caused at least USD760 billion in health expenditure in 2019. Most of the diabetic patients were between 40 and 59 years old (1). Furthermore, by using social media as a supporting tool for the main intervention is an advantage for improving healthcare outcomes in diabetic patients (2). In Malaysia, social media users were about 24.6 million with the most favoured social media platform were Facebook (97.3%), Instagram (57.0%), YouTube (48.3%), Google+ (31.3%), Twitter (28.3%) and LinkedIn (13.3%) (3). The internet and social media were used to access for general information, topics on education, to communicate and connect with another person who shares the same illness, to provide or receive support, and exchange knowledge on self-management and diabetic awareness (4).

Many studies have found that majority of the participants used the internet as their resources for health-related information and closely adhered to health-related information provided by healthcare professionals (5). The side effects of the medications, signs, symptoms and diagnoses of the disease are popular focus for the participants (5).

In this study, predominant of participants (89.8%) used WhatsApp, followed by Facebook (58.6%) and Twitter (42.3%) (6). Regardless at any time or any locations, the media allows the users to share unrestricted and unattended access to unfiltered information (6). The media consists of information which is user-developed content and permits the three-way communication (7). The patients also shared their experiences, feelings and health-related information, exchanged ideas about health-related issues and sought for social and emotional support from their relatives and friends via Facebook, WhatsApp and Twitter (8, 9). The proper usage of the social media could contribute to significant benefits and assist the patients (10). For instance, a researcher from Saudi Arabia found awareness on health-related issues was raised via Twitter for health-related promotion strategies (11). Some studies had shown a positive effect of media usage on healthcare, such as mental health, self-management, physical fitness programme, and behaviour-related outcomes (12–15).

For an organisation, this would benefit by lessening the burden on readmission cases. Furthermore, the healthcare provider could identify the best social media preference to engage with the public that would likely improve healthcare outcomes for the patients to gain knowledge from the shared information on social media by the health professional.

However, there is little known about the social media preference reflected by health-related information received in the media amongst diabetic patients in Malaysia. According to a study conducted in the Dutch language, there was limited data available on the percentage of diabetic patients who used the social media, and their preferences to access health-related information (5). On the other hand, previous studies were not focused on specific diseases such as diabetes mellitus, hypertension and cancer patients. Furthermore, previous research also did not study the reliability of the social media used and the quality of information (5). Therefore, a study was required to be conducted for determining the media preference reflected by health-related information received on social media amongst diabetic patients in Hospital Canselor Tuanku Muhriz (HCTM), Kuala Lumpur, Malaysia.

## Methods

### Study Design, Setting and Sample

This research was conducted by using a cross-sectional design. The study population consisted of 376 diabetic patients who came for an appointment in January 2019 at Medical Clinic 2, HCTM. Eligible participants had to be Type 1 or Type 2 diabetes mellitus (T1DM or T2DM) who were able to understand the Malay or English language. Those who were non-internet users and patients diagnosed with gestational diabetes mellitus were excluded from the study. The sample size was measured for 1 month by using a formula by Krejcie and Morgan (16) with an estimation of 300 population samples. A pilot study was conducted on 30 participants via WhatsApp by using Google documents, which was not applied in the actual research. The Cronbach’s alpha value for the relationship between socio-demographic data and media preference was 0.783, while the relationship between health-related information and media preference was 0.813, and the relationship between frequency of media usage and media preference was 0.713, respectively. A Cronbach’s alpha value of 0.70 and above was considered acceptable or good (17).

### Instruments

This study used a self-reported questionnaire modified from studies conducted by Van de Belt et al. (5) and Gabarron et al. (18). The questionnaire consisted of two parts: i) Part A: socio-demographic data and ii) Part B: use of the internet and media for health-related information. The questionnaire was modified specifically for the Malaysian population and available in both the English and Bahasa Malaysia languages. Part A consisted of five socio-demographic data, whereby the researchers obtained information regarding gender, age, occupational status, levels of education and types of diagnosed diabetes. Part B consisted of eight questions on health-related information, frequency of media usage, and media preference which were measured by using the internet and social media. Additionally, there were four multiple choice questions, and four normal questions in Part B.

### Data Collection Process

Data collection was conducted in 3 weeks. The process began with the identification of potential respondents following the criteria. Thereafter, the respondents were given an information sheet and written consent to be completed if the respondents agreed to participate in the study. The questionnaires were distributed and must be completed before the respondents leave the clinic. All the submitted questionnaires were checked by the researchers to retrieve any incomplete questionnaires.

### Data Analysis

Data for this study were analysed by using the SPSS software version 26.0 for Windows. Descriptive analyses were performed to outline socio-demographic data characteristics, media preference, health-related information and frequency of media usage. Inferential analyses were performed at the significant level of *P* < 0.005. Chi-square test was utilised to identify the relationship between socio-demographic data and media preferences, to identify the relationship between health-related information and media preferences, and to identify the relationship between the frequency of media usage and media preferences.

## Results

### Respondents’ Socio-Demographic Characteristics

Table 1 illustrates the respondents’ sociodemographic characteristics for this study. On average, the respondents were 52.9% males (*n* = 92), and 47.1% females (*n* = 82). The level of education amongst the respondents was mainly secondary school level (*n* = 70, 40.2%). In terms of occupational status, most of the respondents were unemployed (*n* = 64, 36.8%). Majority of the respondents were T2DM (*n* = 128, 73.6%). As for the age, about half of the respondents were middle-aged (45–64 years old) (*n* = 96, 55.2%).

### Media Preference

As reflected in Table 2, most of the respondents used WhatsApp and Facebook to share and search for health-related information. Most of the respondents preferred to use WhatsApp to share and search for health-related information which exceeded 73.6% (*n* = 128). Furthermore, a majority of the respondents, 69.8% (*n* = 118) preferred to use Facebook as a platform of social media to share and search for health-related information. Based on the results, diabetic patients preferred using WhatsApp (73.6%) as their social media platform to obtain and share health-related information. This is similar to a study by syndrome (19) amongst diabetic population in Saudi Arabia. These findings were also supported by the Malaysian Communications and Multimedia Commission 2018, whereby it stated that ‘online communication application that is most preferred amongst Malaysians is WhatsApp, whereby 98.1% have WhatsApp accounts’.

### Relationship between Socio-Demographic Data and Media Preference

#### Relationship between Gender and Media Preference for Searching or Sharing Health-Related Information

Based on Table 3, the media preference related to gender was not significant, *P* = 0.067. The male respondents preferred to use WhatsApp, while the female respondents preferred to use both Facebook and WhatsApp as the social media platform to search or share health-related information. This finding corresponds with the studies conducted by Kamis et al. (20) and Terschüren et al. (21), that showed men were more likely to accept telemonitoring than women. Nevertheless, this finding is contradictory to the findings published by Iftikhar and Abaalkhail (6), and Van de Belt et al. (7), whereby those who completed the questionnaires comprised mainly of women.

### Relationship between the Level of Education and Media Preference

As seen in Table 4, the results indicated the social media preference related to the level of education was not statistically significant, *P* = 0.027. Respondents with an education up to secondary school level or lesser showed preference towards WhatsApp for searching and sharing health-related information. Undergraduates, on the other hand, were likely to choose Facebook as their preferred social media. The majority of postgraduates preferred to use WhatsApp to share or search for health-related information online. This study was consistent with the studies by Sarkar et al. (22) and Song et al. (23) which indicated that education is not associated with the use of e-Health. However, this finding is contradictory with a previous study conducted by Iftikhar and Abaalkhail (6). According to Iftikhar and Abaalkhail (6), patients who had attained postgraduate university degrees were more likely to verify the credibility of information received via the social media platform.

### Relationship between Occupational Status and Media Preference

The results indicated that the media preference correlated to occupational status and the media preference was not statistically significant, *P* = 0.039. The other details are described in Table 5. The unemployed diabetic patients preferred to use WhatsApp to search or share health-related information, whilst, the government servants preferred to use both Facebook and WhatsApp to share and search for health-related information. Furthermore, the private sectors also showed the same preference for Facebook and WhatsApp as a social media platform to search and share health-related information. Meanwhile, most self-employed respondents preferred to use Facebook for searching and sharing health-related information.

### Relationship between the Type of Diabetes Mellitus and Media Preference

Table 6 describes the media preference related to the type of diabetes mellitus was not significant *P* = 0.009. T1DM preferred to use Facebook, while T2DM preferred to use WhatsApp for searching and sharing health-related information. The results showed that most of the diabetic patients in HCTM were diagnosed as T2DM (66.9%). This finding is consistent with a study conducted by Nelakurthi et al. (24) that presented nearly all the users of social media were T2DM (65%). In contrast, the largest number of participants was T1DM, who used social media to obtain information (95%) (19).

### Relationship between Age and Media Preference

The results indicated that the social media preference was statistically related to age, *P* = 0.002, as demonstrated in Table 7. The Cramer’s V was 0.15, which indicated a medium effect size. Diabetic patients aged between 18 and 44 years old chose Facebook as the preferred social media to share and search for health-related information. On the other hand, diabetic patients aged between 45 and 64 years old preferred to use WhatsApp to share and search for health-related information. Likewise, patients aged above 64 years old also preferred to use WhatsApp to share and search for health-related information.

Most of the respondents (59.0%) who used social media to access health-related information, were aged between 45 and 64 years old. This coincides with a previous study conducted in the Dutch language using questionnaire that was created by Van de Belt et al. (5), whereby 47.4% social media users were 45–64 years old. Meanwhile, this contradicts with a previous study on age, which stated that the participants in the dotage groups were more often attentive to the telemedical devices than the adolescent (25). Other studies showed that the older user groups were more dedicated to their health goals and potentially were more enthusiastic about participating in daily self-management activities (26). However, there was a small amount of discrepancies in the current sample of certain age groups, and unequal representation across groups which limits the ability to draw direct comparisons (27).

### Relationship between Health Information and Media Preference

Table 8 shows the media preference was not significantly correlated to the health-related information which was searched or shared on social media, *P* = 0.094. The results showed most diabetic patients searched or shared the information about the medication and side effects (71.3%), followed by self-care (66.7%), and symptoms (55.2%). Similarly, a previous study by Van de Belt et al. (5) showed that the most popular topics searched online were side effects of medication, and symptoms. Other than that, lack of health education from healthcare providers regarding medication side effects could be the answer to why patients are searching for health-related information, especially regarding medication and side effects. As evidence, lack of awareness regarding drug interaction is an issue that warrants further intervention by increasing the knowledge of the public, to prevent adverse events from the drug. Hence, healthcare providers should make an effort to educate the public on this deficiency in knowledge (28).

### Relationship between Frequency of Media Usage and Media Preference

Table 9 illustrates the media preference was not significantly correlated to the frequency of social media usage, *P* = 0.094. The result showed that diabetic patients rarely searched for health-related information online (42.2%), while only 11.0% searched for information on social media once every two to three weeks. This study contradicts with Van de Belt et al. (5), whereby the Dutch population used social media to search for health-related information at least once a year (92.0%), but used the media at least every month (24.4%). However, diabetic patients preferred to use WhatsApp weekly to search for health-related information (43.5%). This finding is contradictory with a previous study by Gabarron et al. (18) who found that Instagram was more frequently used by participants who were less than 18 years old.

### Strengths and Limitations

Overall, the study instruments were the strength for this study which could reflect the objectives required precisely. Furthermore, the sample of participants was more specific, whereby it focused on target diabetic patients (commonly diagnosed and increasing rapidly).

There were limitations to be considered in this study. Due to the current worldwide COVID-19 pandemic, the duration allocated for data collection was shortened. The number of diabetic patients at the clinic decreased and the guideline of one-metre physical distancing restricted the targeted response rate. Moreover, the response rate was limited for most diabetic patients did not use the internet in their daily lives.

## Discussion

The study was conducted between several socio-demographic variables that include gender, level of education, occupational status, types of diabetes mellitus, age, health-related information search and frequency of media usage. The results obtained indicated that WhatsApp (73.6%) was the most used social media platform by almost all variables followed by Facebook (67.8%), Instagram (18.4%) and Twitter (17.2%).

In the gender category, men preferred to use WhatsApp, whereas women preferred to use both WhatsApp and Facebook. All levels of education preferred to use WhatsApp except the undergraduate levels preferred to use Facebook. In the occupational status category, the unemployed preferred to use WhatsApp which was different with the self-employed who preferred to use Facebook. Other occupational status preferred to use both Facebook and WhatsApp. In the age category, all ages preferred to use WhatsApp except for patients between the age of 18 and 44 years old preferred to use Facebook. The three topics that patients searched the most were medication, side effects and self-care. Through these studies, it was found that the patients rarely searched or shared health-related information using online media.

Based on the information obtained from the results, a guideline on the best online tools for health communication and future proposal could be developed. The best application is WhatsApp, followed by Facebook. Therefore, healthcare providers should collaborate with the diabetic patients to build a proper and trusted page, especially through WhatsApp and Facebook for media communication. The contents should involve full information about diabetes care and management, especially medication and side effects, other patient’s experience, symptoms and self-care for patients’ references, and post-clinic appointments.

Besides, a person in-charge should be appointed to perform a special task, such as to communicate with patients through the media platform. In a nutshell, the healthcare providers should also use webinars for health awareness programmes for family members. The method is more practical with the current condition in parallel with the development of increasingly sophisticated technology, whereas other patients in the developed countries are more active through online communities.

## Conclusion

A guideline on the best online tools for health communication has been developed based on media preference on health-related information received amongst diabetic patients in HCTM. The study findings revealed that the preferred social media was WhatsApp, followed by Facebook, Instagram and Twitter for receiving, sharing or searching for health-related information amongst diabetic patients in Malaysia. As a result, the most searched or shared health-related information was about medication and side effects, self-care and symptoms of related diseases. These results could be a guideline for the medical practitioners or researchers to select the best online tools, particularly WhatsApp, to spread health-related information to the diabetic patients and all other patients in Malaysia.

The diabetic patients also rarely used the social media to search for health-related information. Overall, this translates that diabetic patients were not relying much on social media to search for health-related information. However, social media have been an essential media platform to enhance public awareness concerning public health. Therefore, healthcare practitioners must emphasise on health promotion and empower the patients to play an active role in their healthcare outcome. Healthcare providers, especially in the nursing sector, should give special attention to this finding for improvements in conducting healthcare education.

## Figures and Tables

**Table 1:** Socio-demographic data of respondents (*N* = 174)

Characteristics		Frequency	Percentage
Gender	Male	92	52.9
	Female	82	47.1
Education level	No education	2	1.1
	Primary school	4	2.3
	Secondary school	70	40.2
	Undergraduate	54	31.0
	Postgraduate	44	25.3
Occupational status	Unemployed	64	36.8
	Government	58	33.3
	Private	34	19.5
	Self-employed	18	10.3
Diagnosis type	Diabetes Type 1	46	26.4
	Diabetes Type 2	128	73.6
Age	Adult (18–44 years old)	38	21.8
	Middle age (45–64 years old)	96	55.2
	Older age (> 64 years old)	40	23.0

**Table 2:** Media preference, health information and frequency of media usage among people with diabetes (*N* = 174)

Variables		Frequency	Percentage
Media preference	Facebook	118	67.8
	Twitter	30	17.2
	Instagram	32	18.4
	WhatsApp	128	73.6

**Table 3:** Relationship between gender and media preference for searching or sharing for health information

Gender	Media preference										χ^2^	Sig-χ^2^

	Facebook		Twitter		Instagram		WhatsApp		Total			
				
	Frequency	%	Frequency	%	Frequency	%	Frequency	%	Frequency	%		
Male	58	35.8	16	9.9	20	**12.3**	**68**	42.0	162	100.0	1.5	0.6
Female	**60**	41.1	10	6.8	16	**11.1**	**60**	41.1	146	100.0	4	7

Total	118	38.3	26	8.4	36	**28.1**	**128**	41.6	308	100.0		

Note:

* Chi-square test

**Table 4:** Relationship between level of education and media preference for searching or sharing for health information

Level of education	Media preference										χ^2^	Sig-χ^2^

	Facebook		Twitter		Instagram		WhatsApp		Total			
				
	Frequency	%	Frequency	%	Frequency	%	Frequency	%	Frequency	%		
Secondary school or lesser level	46	39.0	6	5.1	10	8.8	**56**	**47.5**	118	100.0	5.87	0.12
Undergraduate	**42**	**42.0**	10	10.0	12	12.0	36	36.0	100	100.0	2.07	0.56
Postgraduate	30	33.4	10	11.1	14	15.5	**36**	**40.0**	90	100.0	3.60	0.31

Total	118	38.3	26	8.4	36	11.7	**128**	**41.6**	308	100.0	7.56	0.27

Note:

* Chi-square test

**Table 5:** Relationship between occupational status and media preference for searching or sharing for health information

Occupational status	Media preference										χ^2^	Sig-χ^2^

	Facebook		Twitter		Instagram		WhatsApp		Total			
				
	Frequency	%	Frequency	%	Frequency	%	Frequency	%	Frequency	%		
Unemployed	36	32.1	12	10.7	14	12.5	**50**	**44.6**	112	100.0	3.33	0.34
Government	**46**	**43.4**	4	3.8	10	9.4	**46**	**43.4**	106	100.0	6.10	0.11
Private	**22**	**36.7**	8	13.3	8	13.3	**22**	**36.7**	60	100.0	2.77	0.43
Self-employed	**14**	**46.7**	2	6.7	4	13.3	10	33.3	30	100.0	1.35	0.72

Total	118	38.3	26	8.4	36	11.7	**128**	**41.6**	308	100.0	9.56	0.39

Note:

* Chi-square test

**Table 6:** Relationship between type of diabetes and media preference for searching or sharing for health information

Type of DM	Media preference										χ^2^	Sig-χ^2^

	Facebook		Twitter		Instagram		WhatsApp		Total			
				
	Frequency	%	Frequency	%	Frequency	%	Frequency	(%)	Frequency	%		
T1DM	38	37.3	10	9.8	18	17.6	36	35.3	102	100.0	6.45	0.09
T2DM	80	38.8	16	7.8	18	8.7	**92**	**44.7**	206	100.0		

Total	118	38.3	26	8.4	36	11.7	**128**	**41.6**	308	100.0		

Note:

* Chi-square test

**Table 7:** Relationship between age and media preference for searching or sharing for health information

Age	Media preference										χ^2^	Sig-χ^2^	Cramer’s V

	Facebook		Twitter		Instagram		WhatsApp		Total				
				
	Frequency	%	Frequency	%	Frequency	%	Frequency	%	Frequency	%			
18 – 44 years old	34	45.9	8	10.9	10	13.5	22	29.7	74	100.0	5.69	0.13	
45 – 64 years old	70	38.4	14	7.7	24	13.2	74	40.7	182	100.0	1.24	0.74	
> 64 years old	14	27.0	4	7.7	2	3.8	32	61.5	52	100.0	11.46	0.01	

Total	118	38.3	26	8.5	36	11.6	128	41.6	308	100.0	14.35	0.02	0.15

Note:

* Chi-square test

**Table 8:** Relationship between health information and media preference for searching or sharing for health information

Health information	Media preference						

	Facebook	Twitter	Instagram	WhatsApp	Total	χ^2^	Sig-χ^2^
Second opinion	56	20	26	54	156	2.29	0.52
Medication and/or side effect	86	22	30	**98**	236	1.77	0.62
Other patient’s experiences	66	16	22	74	178	1.58	0.66
Specific diagnoses	54	20	22	56	152	1.39	0.71
Therapy or treatment	66	14	24	66	170	1.04	0.79
Symptoms	**78**	22	26	70	196	0.87	0.83
Health problems	66	18	24	62	170	0.21	0.98
Self-care	82	18	32	**92**	224	2.20	0.53
Research on diabetes	54	20	24	44	142	4.77	0.19

Total	608	170	230	616	1624	14.37	0.94

**Table 9:** Relationship between frequency of media usage and media preference for searching or sharing for health information

Frequency	Media preference						

	Facebook	Twitter	Instagram	WhatsApp	Total	χ^2^	Sig-χ^2^
Once daily	20	6	6	20	52	0.86	0.81
Once every two to three days	12	4	4	14	34	0.59	0.85
Weekly	38	4	10	40	92	3.10	0.38
Rare	48	12	16	54	130	0.35	0.95

Total	118	26	36	128	308	3.62	0.94

## References

[1] International Diabetes Federation (2019). International diabetes atlas. International Diabetes Federation.

[2] Gabarron E, Arsand E, Wynn R (2018). Media use in interventions for diabetes: rapid evidence-based review. J Med Internet Res.

[3] Malaysian Communications and Multimedia Commissions (2018). Internet users survey 2018: statistical brief number 23.

[4] Alzahrani A, Alanzi T (2019). Social media use by people with diabetes in Saudi Arabia: a survey about purposes, benefits and risks. Diabetes Metab Syndr Obes.

[5] Van de Belt TH, Engelen LJLPG, Berben SAA, Teerenstra S, Samsom M, Schoonhoven L (2013). Internet and media for health-related information and communication in healthcare: preferences of the Dutch general population. J Med Internet Res.

[6] Iftikhar R, Abaalkhail B (2017). Health-seeking influence reflected by online health-related messages received on media: Cross-sectional survey. J Med Internet Res.

[7] Van de Belt TH, Engelen LJLPG, Berben SAA, Schoonhoven L (2010). Definition of health 2.0 and medicine 2.0: a systematic review. J Med Internet Res.

[8] Antheunis ML, Tates K, Nieboer TE (2013). Patients’ and health professionals’ use of media in healthcare: motives, barriers and expectations. Patient Educ Couns.

[9] Greene JA, Choudhry NK, Kilabuk E, Shrank WH (2011). Online social networking by patients with diabetes: a qualitative evaluation of communication with Facebook. J Gen Intern Med.

[10] Sawesi S, Rashrash M, Phalakornkule K, Carpenter JS, Jones JF (2016). The impact of information technology on patient engagement and health behavior change: a systematic review of the literature. JMIR Med Inform.

[11] Albalawi Y, Sixsmith J (2017). Identifying Twitter influencer profiles for health promotion in Saudi Arabia. Health Promot Int.

[12] Kear T, Harrington M, Bhattacharya A (2015). Partnering with patients using media to develop a hypertension management instrument. J Am Soc Hypertens.

[13] Laranjo L, Arguel A, Neves AL, Gallagher AM, Kaplan R, Mortimer N (2014). The influence of social networking sites on health behavior change: a systematic review and meta-analysis. J Am Med Inform Assoc.

[14] Shepherd A, Sanders C, Doyle M, Shaw J (2015). Using media for support and feedback by mental health service users: thematic analysis of a twitter conversation. BMC Psychiatry.

[15] Williams JP, Schroeder D (2015). Popular glucose tracking apps and use of mHealth by Latinos with diabetes: review. JMIR Mhealth Uhealth.

[16] Krejcie RV, Morgan DA (1970). Determining sample size for research activities. Educ Psychol Meas.

[17] Nunnally JC (1978). Psychometric theory.

[18] Gabarron E, Dorronzoro E, Bradway M, Rivera-Romero O, Wynn R, Årsand E (2018). Preferences and interests of diabetes media users regarding a health-promotion intervention. Patient Prefer Adherence.

[19] Alzahrani SA, Alanzi T (2019). Media use by people with diabetes in Saudi Arabia: a survey about purposes, benefits and risks. Diabetes Metab Syndr Obes.

[20] Kamis K, Janevic MR, Marinec N, Jantz R, Valverde H, Piette JD (2015). A study of mobile phone use among patients with noncommunicable diseases in La Paz, Bolivia: implications for mHealth research and development. Global Health.

[21] Terschüren C, Mensing M, Mekel OC (2012). Is telemonitoring an option against shortage of physicians in rural regions? attitude towards telemedical devices in the North Rhine-Westphalian health survey, Germany. BMC Health Serv Res.

[22] Sarkar U, Piette JD, Gonzales R, Lessler D, Chew LD, Reilly B (2008). Preferences for self-management support: findings from a survey of diabetes patients in safety-net health systems. Patient Educ Couns.

[23] Song L, Tatum K, Greene G, Chen RC (2017). e-Health literacy and partner involvement in treatment decision making for men with newly diagnosed localised prostate cancer. Oncol Nurs Forum.

[24] Nelakurthi AR, Pinto AM, Cook CB, Jones L, Boyle M, Ye J (2018). Should patients with diabetes be encouraged to integrate media into their care plan?. Future Sci.

[25] Whittemore R, Jaser SS, Faulkner MS, Murphy K, Delamater A, Grey M (2013). Type 1 diabetes ehealth psychoeducation: Youth recruitment, participation, and satisfaction. J Med Internet Res.

[26] Goyal S, Morita PP, Picton P, Seto E, Zbib A, Cafazzo JA (2016). Uptake of a consumer-focused mhealth application for the assessment and prevention of heart disease: the <30 days study. JMIR Mhealth Uhealth.

[27] Shaffer-Hudkins E, Johnson N, Melton S, Wingert A (2014). Social media use by individuals with diabetes. Int J Commun Health.

[28] Mohamed IMA, Gunasekaran K, Kadirvelu A, Gurtu S, Sadasivan S, Kshatriya BM (2013). Self-medication: awareness and attitude among Malaysian urban population. Int J Collab Res Intern Med Public Health.

